# How and why animal welfare concerns evolve in developing countries

**DOI:** 10.1093/af/vfac082

**Published:** 2023-02-23

**Authors:** Martin Parlasca, Isabel Knößlsdorfer, Gezahegn Alemayehu, Rebecca Doyle

**Affiliations:** Center for Development Research (ZEF), University of Bonn, Bonn, Germany; Agricultural Economics and Rural Development, University of Göttingen, Göttingen, Germany; International Livestock Research Institute, Addis Ababa, Ethiopia; International Livestock Research Institute, Addis Ababa, Ethiopia; The Jeanne Marchig International Centre for Animal Welfare Education, The University of Edinburgh Royal Dick School of Veterinary Studies, Roslin, UK

**Keywords:** animal welfare, consumer preferences, food labeling, Global South

**Implications**

- In developing countries, animal welfare concerns do not receive the same recognition as they do in higher-income countries, from policy and law, through to consumer awareness and purchasing options.
- While traditional farmers often have close bonds with their animals, knowledge and action gaps often limit more animal-friendly production.
- In some developing countries, livestock production has already largely commercialized and intensified. In these countries, citizens are becoming increasingly aware and sensitive to animal welfare issues, but animal welfare does not yet affect purchasing decisions.
- Future scenarios with higher animal welfare are possible, but will require joint efforts by various stakeholders in the livestock sector.
- Overall, much more research on animal welfare perceptions in developing countries is needed.

## Introduction

**Figure 1.**
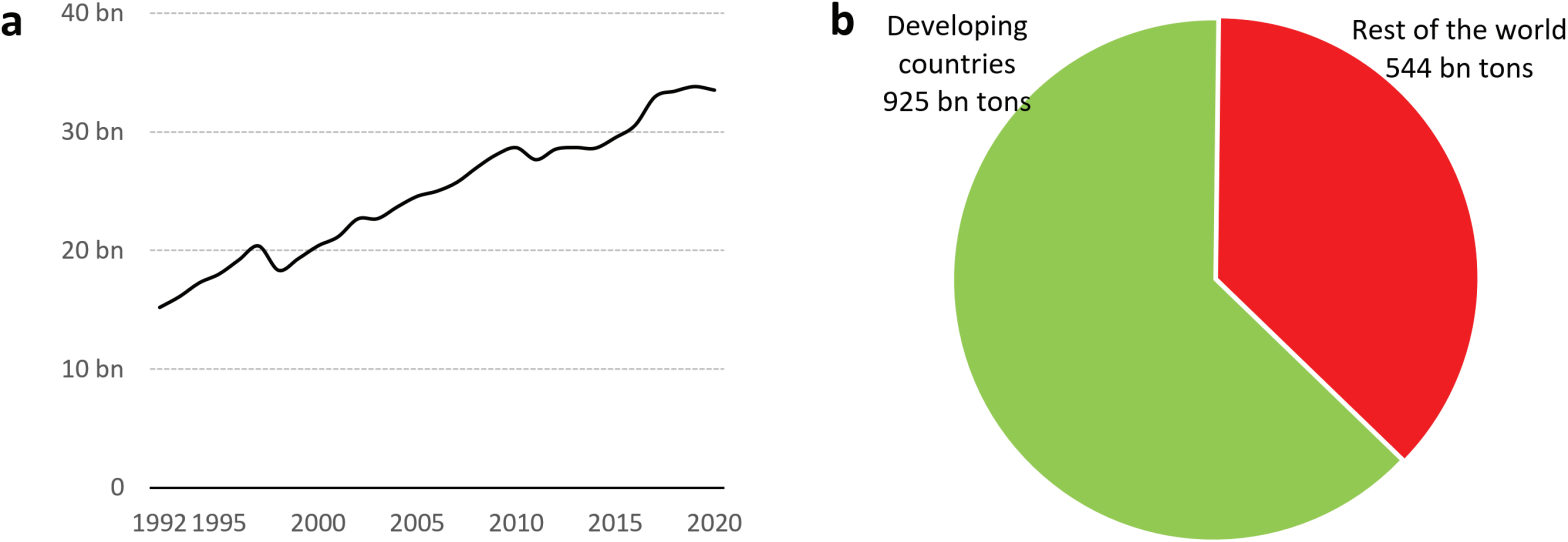
(a) Livestock counts in developing countries. Measured as the number of live animals at a single point in any given year. (b) Total production volumes of animal-source foods in 2020. Source: FAO statistics.

Livestock make several important contributions to healthy diets and livelihoods. This is especially true in developing countries where otherwise nutritious food options are limited. To ensure the sustainability of food systems, production of animal-source foods must also align with societal views regarding farm animal welfare (Parlasca and Qaim, 2022). Even though most farm animals live in developing countries and most animal-source foods are produced there (see Figure 1), research on societal perceptions regarding farm animal welfare has a strong focus on high-income countries. Much less is known about the topic from developing countries’ perspectives (Clark et al., 2016).

The lack of attention given to animal welfare concerns in this context may be explained by the fact that per capita consumption of animal-source foods is relatively low in developing countries, although there are some exceptions such as Mongolia, Argentina, or Brazil (Parlasca and Qaim, 2022). Additionally, research in high-income countries has shown that demand for animal welfare is strongly correlated with wealth and education (Lagerkvist and Hess, 2011). Low consumption of animal-source foods and the low-income levels in developing countries may, therefore, indicate a low effective demand for farm animal welfare among large parts of the population in these countries. In some cultures, traditional customs and beliefs may further reduce the priority assigned to the topic (Ndou et al., 2011). Lastly, prominent animal welfare issues such as confinement in small spaces or limited climate and floor variation are mainly associated with industrialized and intensive animal production. Such systems were in the past mostly found in developed countries rather than developing countries (Steinfeld et al., 2006).

Yet, livestock production in developing countries is not free of farm animal welfare problems. Production of animal-source food in developing countries is gradually converging with high-income countries. Even though extensive and subsistence livestock production is still common in various countries, capital intensity and mechanization of animal-source food production increase in most places (Steinfeld et al., 2006). With that, several farm animal welfare issues are gradually becoming prominent as well (Jankielsohn, 2015). In addition to changes in production systems, people’s willingness and ability to pay for products with higher levels of animal welfare may also slowly rise in developing countries due to economic growth, higher standards of living, and the increasing role of social media campaigns (Shaheer et al., 2021). Overall, animal welfare topics, therefore, are likely to gradually become more important for the civil society, regulatory institutions, and livestock producers in developing countries.

Up till now, however, the body of scientific evidence in this area remains very thin. Animal welfare perceptions were recently reported to have gained attention in some developing countries in South America, as well as in China or Mexico (von Keyserlingk and Hötzel, 2015; Carnovale et al., 2021; Estévez-Moreno et al., 2022), but related research is still sporadic for many other developing countries, especially in Africa and South Asia (Sinclair et al., 2022). Most of the insights presented in this article come from case studies and, therefore, may not be able to fully account for regional differences and nuances. Nevertheless, we try to provide an overview over what we currently know about perceptions of animal welfare in different societies across the developing world and to discuss possible scenarios on how the state of animal welfare could evolve in the future. To do so, we first define animal welfare. We then present important drivers of animal welfare perceptions in developing countries. We then offer a short outlook on how awareness of animal welfare can increase in developing countries and draw final conclusions.

## What is farm animal welfare?

Public discussions on farm animal welfare emerged in high-income countries during the second half of the 20th century. Since then, meaning and interpretation of the term have continuously evolved and exhibit significant regional differences. The meaning and interpretation of what constitutes good animal welfare and the relevance that is assigned to it are not static parameters, but complex and multifaceted issues shaped by cultural, social, economic, ethical, religious, and political dynamics.

In this article, we define farm animal welfare as a state of both physical and mental health of farm animals. It consists of three pillars: animal health, natural living, and affective states (Fraser et al., 1997). Farm animal welfare therefore requires that animals are physically healthy, that they have the opportunity to live a reasonably natural life, and that animals have minimal negative psychological states and at least some positive psychological states.

This definition of animal welfare based on the three pillars does not have a direct pendant in many societies of developing countries (Sinclair and Phillips, 2018; Doyle et al., 2019; Carnovale et al., 2021; Chen and Weary, 2021). In certain societies, people may understand animal welfare to mean only the absence of physical suffering, in others, animal welfare includes animals’ mental states, environments, health, nutrition, and behaviors. Even though the needs of animals are independent of any cultural context, there can be substantial discrepancies among people’s understanding of animal welfare. An empirical example demonstrating this discrepancy is the fact that even the fundamental recognition that animals can experience pain and have physical and emotional needs (see Marino, 2017) has not yet fully taken hold in various societies, including those in high-, middle-, and low-income countries around the world (Sinclair et al., 2022).

The lack of harmonization with regards to animal welfare definitions and perceptions poses a challenge when contexts are highly diverse. While attitudes toward animal welfare and according animal welfare legislations also vary between and within industrialized countries, much more research has been done in these regions. Perceptions toward animal welfare in developing countries are highly understudied, which complicates an assessment.

Science can and needs to contribute to comprehensive definitions and standardized measurements of farm animal welfare, for example, through further development of methods that objectively measure and verify certain animal welfare aspects. That said, there is a risk that discussions on animal welfare in developing countries solely use “Western” definitions of animal welfare, which may not always be appropriate to the cultural and religious contexts in developing countries (Garcia and McGlone, 2022). For example, anecdotal evidence shows that the term “welfare” can be perceived to reflect a kind of luxury. In low-income environments, it has therefore shown to be problematic to discuss and advocate for higher animal welfare when the term is associated with a state of luxury that people themselves do not have—or believe they do not have (Sinclair and Phillips, 2018). Further harmonization of animal welfare definitions on a global level therefore requires science-based yet culturally appropriate and inclusive approaches.

## Drivers of farm animal welfare concerns

Issues for the welfare of farm animals can occur at several different stages of production. These include on-farm treatment, loading and unloading of animals into transport vehicles, and the transport itself. Mental and physical harm can also occur shortly after birth through separation of the mother/siblings, during lairage, on livestock markets, or during stunning and slaughter. While a comprehensive overview of species and supply chain-specific animal welfare issues go beyond the scope of this article, it is clear that potential animal welfare issues depend on level of intensification of production systems, the human–animal relation, and value chain characteristics such as transportation of live animals (Gallo and Huertas, 2016). Underlying production processes are, therefore, a major factor for the type and severity of animal welfare issues that can, in principle, arise for societies in developing countries.

At the same time, the social attitudes toward animal welfare not only depend on the actual conditions of animals, but also on the interpretations and importance attributed to different animal welfare conditions. Someone who places more weight on the biological functioning aspects of animal welfare may, for example, deem the relative behavioral restrictions of confined and modern pig production as acceptable because of the higher biosecurity and disease control opportunities. Another person, who places more weight on opportunities for natural behavior may be critical of such productions because they can restrict behavioral opportunities such as foraging, or social and maternal behaviors (Fraser, 2008a). Such anthropocentric perspectives of animal welfare, which strongly depend on cultural and societal values, also determine fundamental questions with regards to which animal are accepted as farm animal and food in the first place and which ones are not (Ohl and van der Staay, 2012; Manokara et al., 2021). In the following, we highlight some of the most important factors that influence animal welfare perceptions in developing countries.

### Level of intensification of production systems

Animal welfare concerns in scientific and public debates mostly relate to commercial intensive production systems (Grethe, 2017). Such systems are characterized by large numbers of animals that are confined into buildings or feedlots and typically involve grain and other concentrated diets for animal feed (Fraser, 2008a). Commercial intensive production is, hence, much more common for farms producing pig, poultry, and egg products, and less so for ruminants such as cattle or sheep and goats. Besides high concentration of animals, intensification of animal-source food production also involves a focus on cost-cutting, which typically restricts opportunities for animals to move around, reduces staff time per animal, and minimizes certain necessities such as bedding (Fraser, 2008a).

**Figure 2.**
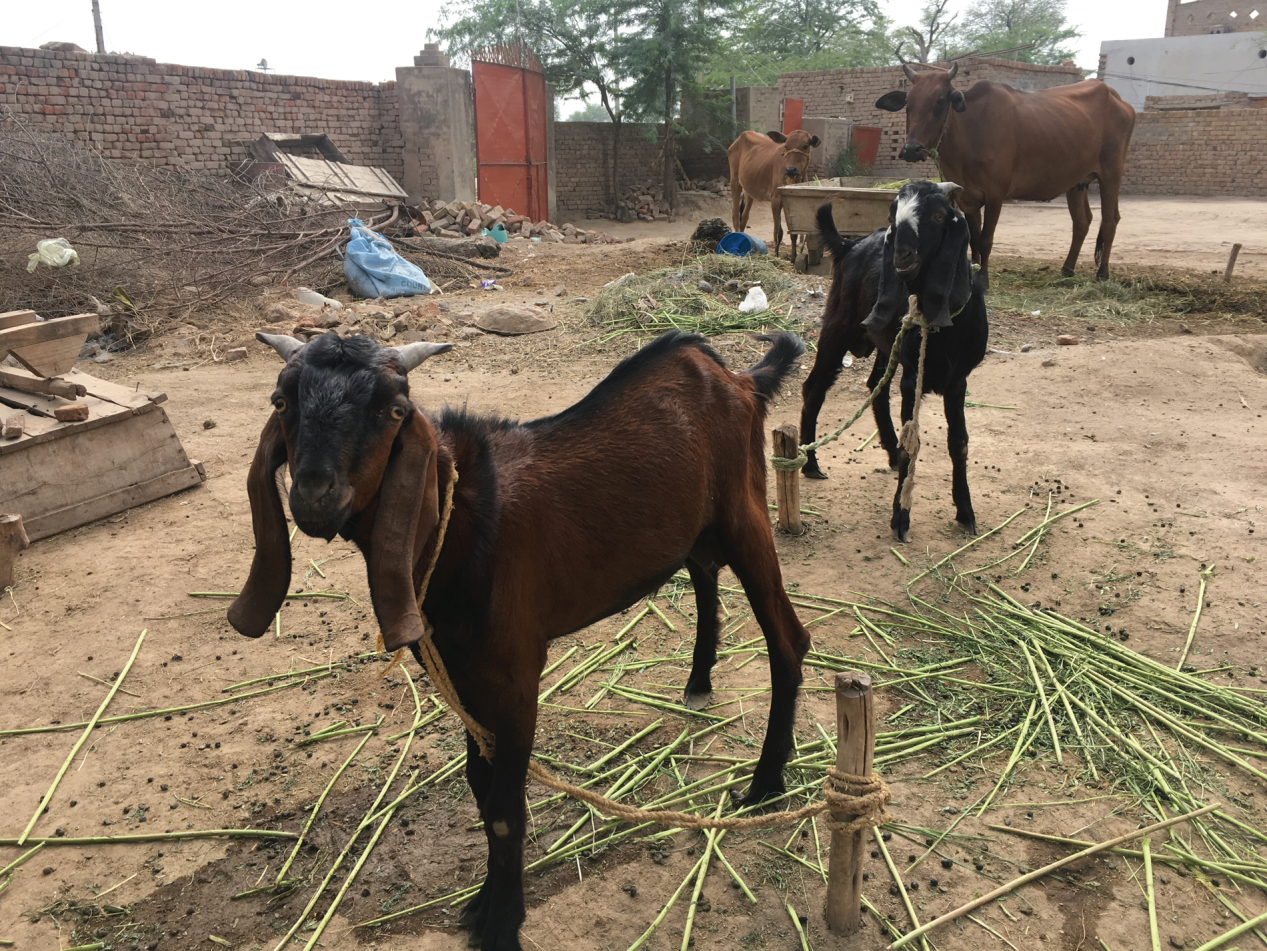
Traditional village farming setting in Saihwahl, Punjab, Pakistan. Livestock are both stall-fed and free-grazed throughout the course of the day (photo credit: Rebecca Doyle).

In high-income countries, production of animal-source foods coming from pigs and poultry is almost entirely intensive. This is not (yet) the case for several developing countries, where backyard systems, as those shown in Figure 2, are still common. In Africa 57% of pork and 13% of poultry comes from backyard production systems, while grazing systems for cattle and small ruminants are still common in both developing and developed countries (Parlasca and Qaim, 2022).

**Figure 3.**
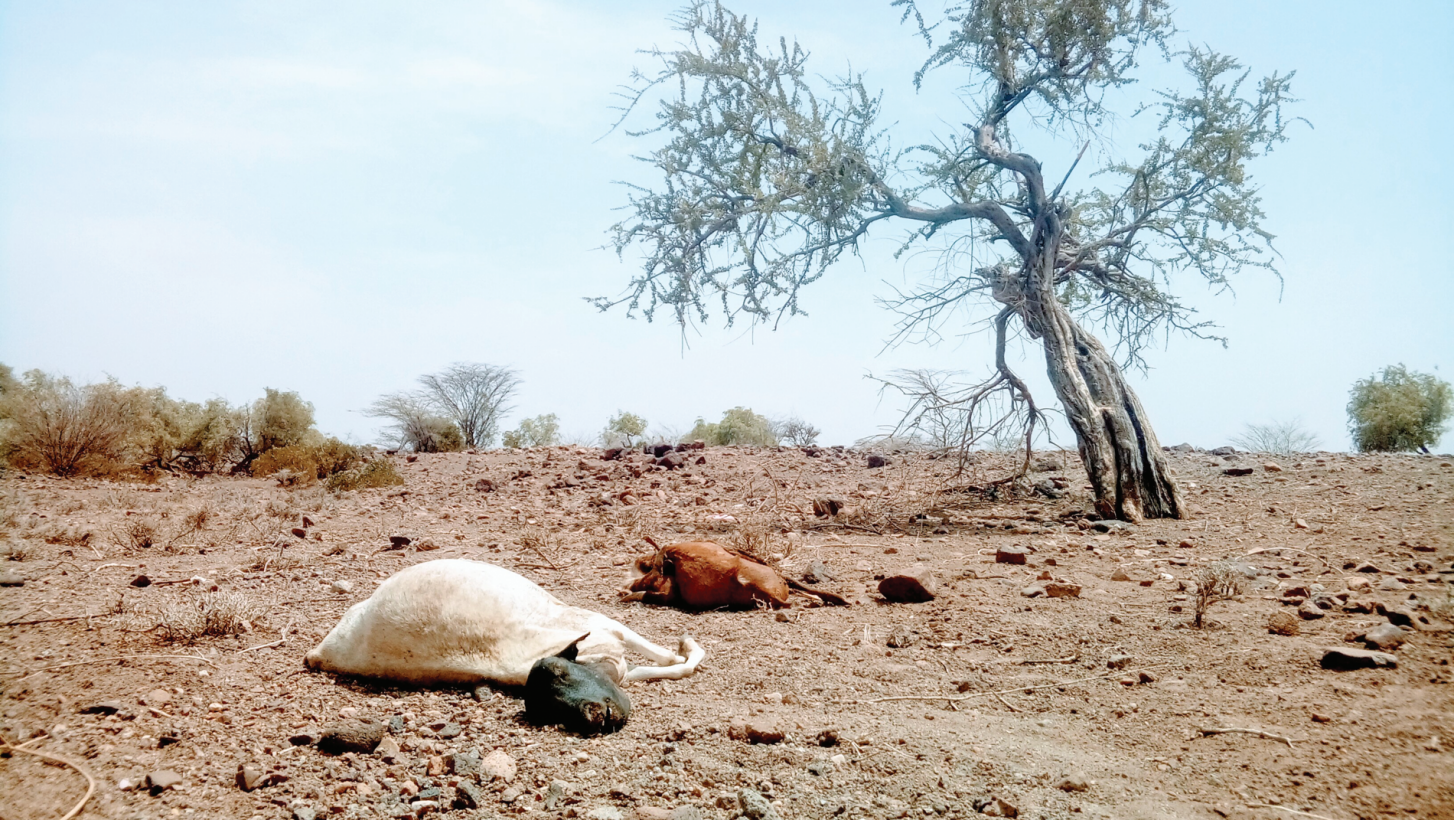
Animals perished during a severe drought near Kokuro, Turkana, Kenya (2017) (photo credit: Martin C. Parlasca).

The welfare of animals kept in extensive systems is oftentimes perceived as better compared to those of animals produced in intensive systems, especially with regards to affective states and natural living (Clark et al., 2016). Yet, extensive systems can also cause some animal welfare issues. Animals may be exposed to heat stress, hunger, or the presence of predators (Temple and Manteca, 2020). Mortality and morbidity, which signal serious animal welfare problems, remain common across large parts of the developing world (Rault et al., 2022) (see Figure 3). Whether extensive systems are generally preferable over intensive systems regarding animal welfare involves value judgments and trade-offs between different animal welfare dimensions. It can, therefore, not easily be answered by science alone. Still, there is little doubt that each type of production system has welfare challenges that need to be addressed.

The complexity and multidimensionality of animal welfare are rarely accounted for in traditional livestock systems in developing countries (Qekwana et al., 2019). That said, many farmers in traditional livestock systems do acknowledge their animals’ feelings of sadness or happiness. Farmers attribute happiness of animals, which closely corresponds to animal welfare, predominantly to the availability of sufficient feed and water. Good health and behavioral needs such as freedom to graze or gentle touch during handling are often perceived as relevant for animal happiness as well (Doyle et al., 2019). Given the importance of livestock for food and income generation, animals can take a high priority in traditional farming systems. Animals may be kept longer than is economically justified and care for animals is sometimes even perceived as higher than those for family members (Qekwana et al., 2019).

Still, traditional livestock keepers in developing countries rarely view animal welfare as a goal in itself. When associated positively with animal productivity and food quality, good animal welfare is viewed as acceptable and achievable. Improvements in animal welfare become particularly difficult when trade-offs between productivity and welfare goals exist (Cardoso et al., 2016). While animal welfare is described as a responsibility of animal owners (Doyle et al., 2019), animal owners’ practices do not always align with this view. Hitting and aggressive animal handling, rudimentary castration practices, hoof overgrowth, and misuse of antibiotics are still commonly found in many livestock production systems (Asebe and Gelayenew, 2015). Traditional and extensive production systems in developing countries are, therefore, affected by knowledge and practice gaps, as well as significant constraints in the external operating environment (Wambui et al., 2018; Doyle et al., 2021).

For consumers, animal welfare conditions may oftentimes not be easily observed or verified. Asymmetric information regarding animal welfare can, therefore, lead to a market failure when people want to purchase meat or dairy products with high levels of animal welfare (Grethe, 2017). However, in traditional livestock systems, consumers often also produce significant parts of their food. This is true especially for pastoralists, for whom meat, milk, and animal blood can be the main component of the diet, but also applies for many other smallholder farmers in developing countries at least to some degree. The physical proximity of consumers to farm animals and firsthand information about animals’ living conditions means that they are able to directly assess and sometimes even influence the level of animal welfare associated with their consumption of animal-source foods. Animal welfare in local traditional production systems, which are not yet so strongly decoupled from consumption, is thus likely to be less affected by issues related to asymmetric information compared to more industrial production systems.

**Figure 4.**
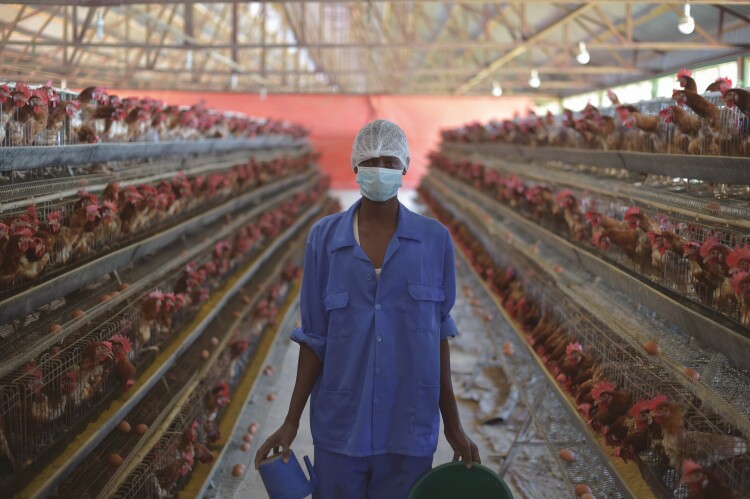
Industrial poultry farm in Mogadishu, Somalia. Among the first farms of its kind in the country, this production unit can keep over 30,000 chickens (photo credit: The African Union Mission in Somalia/Tobin Jones).

Even though extensive and smallholder farming systems are still common in most developing countries, the production of animal-source food intensifies in most places. Much of the growth in animal-source food production that has taken place in the last decades (see Figure 1) has been achieved through the adoption of high-input farming systems that are similar to those in developed countries (see Figure 4).

**Figure 5.**
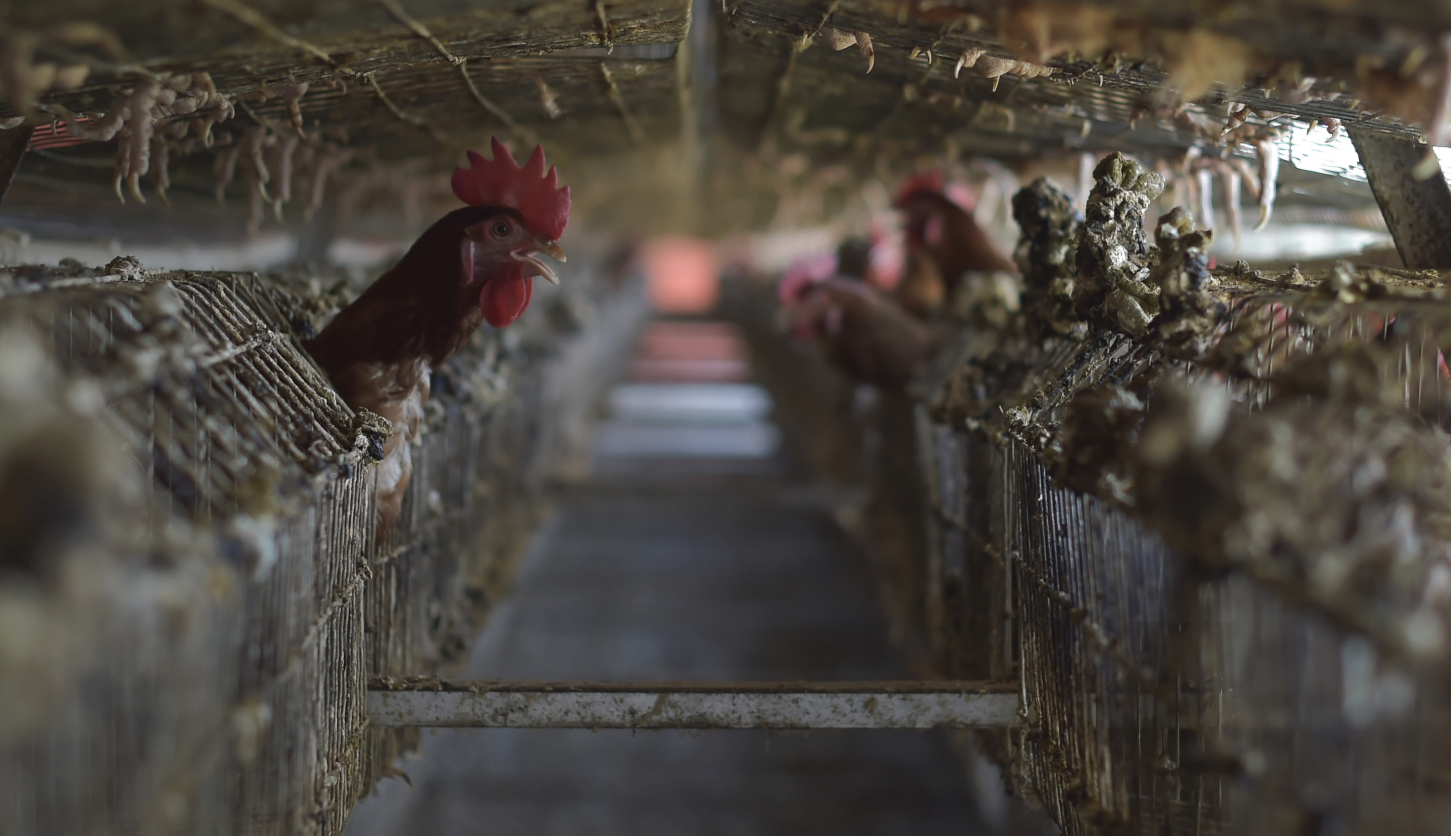
Industrial poultry farm in Mogadishu, Somalia. High stocking densities for layer hens can create animal welfare issues (photo credit: The African Union Mission in Somalia/Tobin Jones).

Intensive systems for pig and poultry production now clearly dominate other production systems in some regions, most notably South America and China (Parlasca and Qaim, 2022). Such commercial intensive systems can be accompanied with unique constraints to animal welfare, including behavioral and physical restrictions in many situations (see Figure 5). Intensive livestock systems in developing countries may additionally cause animal welfare issues due to a mismatch between animal genetics, which are often bred for environments found in high-income countries, and the environment in which these animals actually live in developing countries (Nielsen and Zhao, 2012).

In commercial intensive systems, farmers/animal caretakers typically do not have the same connectedness to their livestock compared to traditional farmers (Masiga and Munyua, 2005). We hypothesize that some of the animal welfare issues described developed income countries may risk becoming exacerbated in developing countries where consumer, regulatory, and legislative controls around animal welfare are also less present.

In those developing countries where intensive animal production systems are very common, animal welfare is a publicly discussed issue that concerns consumers, producers, and regulatory institutions. Estévez-Moreno et al. (2022) found that a large majority of consumers across six South American countries share the fundamental belief that animal welfare is important. Similar development can be observed in China or Mexico, where animal welfare is receiving increasing attention in the mainstream society (Carnovale et al., 2021; Estévez-Moreno et al., 2021). This indicates that animal welfare considerations are, at least to some degree, part of a universal human value. Still, the relevance of the topic and the vehemence of public discussions about animal welfare in these countries are much lower compared to Western Europe or North America (von Keyserlingk and Hötzel, 2015).

Even in developing countries where the topic of animal welfare is slowly moving on the radar of consumers, animal welfare considerations so far have minimal impacts on actual purchasing decisions. In Brazil, for example, most people agree that animal welfare is generally a relevant topic, yet less than 4% of interviewed consumers consider animal welfare during meat purchases (Bonamigo et al., 2012). The discrepancy between stated preferences about animal welfare and actual purchasing decisions, which is also known as the citizen consumer gap, is a well-researched topic for developed countries and can be explained by social desirability bias, as well as a lack of awareness of or engagement with potential animal welfare issues (Grethe, 2017). A substantial lack of consumers’ knowledge of production processes has been found to be associated with animal welfare perceptions in other South American countries as well as China (Estévez-Moreno et al., 2022). This lack is, therefore, most likely one of the key reasons for the low relevance of farm animal welfare issues during meat purchases.

### Religion and culture

Religious beliefs and cultural traditions can be critical for animal welfare perceptions. The idea that animals should not be used for food or any purpose, for example, is a widespread view of Jains, Buddhists, and many Hindus (Szűcs et al., 2012). Developing countries are rich in cultural and religious diversity. While most of these religions teach respect and compassion for animals and reject unnecessary suffering (Rahman, 2017), certain religious practices, such as animal sacrifices, can also be source of animal welfare issues.

In order to minimize unnecessary pain, the responsibility of slaughtering for such types of slaughter is typically given to skilled people such as elders or well-respected community members. At the same time, animals’ bellowing during traditional sacrifices is an important indicator that the sacrifice has been successful. Stunning, which supposedly reduces stress and pain for the animal before and during slaughter, is therefore often not compatible with traditional beliefs (Qekwana et al., 2019). Although animal sacrifice or ritual slaughter of animals is not uncommon in many developing countries, such activities account for only a small portion of potential animal welfare issues in developing countries. However, it further emphasizes that discussion on animal welfare needs to be sensitive to cultural and religious contexts.

### Rural–urban origin

Animal welfare perceptions can vary between rural and urban citizens (Kendall et al., 2006). Even though not much is yet known about this connection for developing countries, the relationship seems to be ambiguous. Rural pork consumers in Vietnam, for example, were more likely to consider animal welfare issues and care about how pigs were kept on the farm than urban consumers (Smith et al., 2021). Yet, opposite results were found in Mexico, where urban consumers tend to place a higher value on animal welfare (Estévez-Moreno et al., 2021). Differences between rural and urban consumers with regards to animal welfare perceptions are possibly driven by rural consumers’ social and physical proximity to farmers and better knowledge of farm animals’ living conditions (Estévez-Moreno et al., 2021).

## Moving toward higher awareness of animal welfare in developing countries

Livestock production systems, cultures, and ethical viewpoints are diverse across and within developing countries. Still, there is an urgent need for animal welfare to increase in many places. In societies where animal welfare concerns are part of public discussions, consumers are generally supportive of approaches toward more animal welfare. In various South American countries or China, for example, consumers are in favor of animal welfare being part of school curricula and that policies and regulations to improve farm animal welfare are appropriate (Carnovale et al., 2021; Estévez-Moreno et al., 2022).

As of now, legislature regarding animal rights in most developing countries is poor or does not exist at all. With some exceptions such as India, Malaysia, or Mexico, animal welfare legislation in developing countries lags behind those of most developed countries. Protection of farm animals in particular is even less enshrined in legislature (Word Animal Protection, 2022). Animal welfare standards in developing countries face several obstacles. Among Chinese producers, for example, such policies are often only perceived as to threat production and profits (Meng et al., 2012). While governmental roles have been important for the establishment of higher animal welfare standards in many developed countries, it is also questionable whether similar approaches will successfully influence the animal welfare situation in traditional, rural, and informal sectors of developing countries due to difficulties in monitoring and enforcement (Fraser, 2008b).

Relatively higher animal welfare standards in developed countries can also have an influence on animal welfare in developing countries through requirements for exports (Grethe, 2017). Yet, if these higher welfare standards are not widely shared by the population of the exporting country, such requirements are unlikely to have a spillover effects for domestic consumption. Since external incentives are usually not tailored to local environmental and cultural conditions, they will not be enough to achieve a sustainable solution in terms of animal welfare in the longer run (Garcia and McGlone, 2022). Attempts to reach consensus on animal welfare issues involving all key stakeholders in animal agriculture of developing countries, including citizens, farmers, industry, NGOs, and public authorities, are therefore much more likely to achieve sustainable progress (Poletto and Hötzel, 2012; Sinclair and Phillips, 2018).

NGOs have been instrumental in putting animal welfare onto the agenda in developing countries in many ways; working equid charities, including those of the International Coalition of Working Equids, have a significant presence on animal welfare across developing countries. Animal welfare NGOs contributed to efforts to get animal welfare recognized at the United Nations Environmental Agency (UNEA, 2022). They have also evaluated animal welfare globally and push to raise the profile of farming issues to consumers and farmers (e.g., Open Wing Alliance). The role that they will play on leading action on animal welfare in developing countries will continue to be significant (Qekwana et al., 2019).

## Conclusion

Perceptions of animal welfare are important for the sustainability of food systems. While farm animal welfare has received considerable attention among developed countries, much less is known from the perspective of developing countries. In this article, we provided an overview over animal welfare concerns in the developing world.

We showed that the meaning of what constitutes animal welfare is not harmonized on a global level and therefore varies across different contexts. Furthermore, empirical data on animal welfare concerns among consumers and producers in developing countries are limited. Traditional livestock systems are often characterized by close bonds between farmers and their animals, but these systems are not per se free of animal welfare issues. In these systems, knowledge and action gaps tend to hamper the development toward more animal-friendly production. The commercial intensification of livestock production that has and is occurring in several developing countries creates new animal welfare issues that can be exacerbated by limited consumer expectations and legislative controls. In countries with largely intensified production, such as China, Mexico, or Brazil, citizens are becoming increasingly aware and sensitive to animal welfare issues. However, even in these countries, animal welfare plays a small role when it comes to actual purchasing decisions. Given the various animal welfare issues that exist in most parts of the developing world, improvements in animal welfare are needed. Yet, the pathways to such scenarios are difficult. Future scenarios with higher animal welfare will require joint efforts by various stakeholders in the livestock production sector.

Generally, data on the dynamics of animal welfare concerns in developing countries are still very thin, especially for least developed countries. Most of the insights presented in this article come from case studies and therefore may not be able to fully account for potential regional differences and nuances. Segmentation of animal welfare perceptions has shown to be important for citizens in South America (Estévez-Moreno et al., 2022), similar aspects are therefore certainly relevant for other countries as well. Research on animal welfare perceptions using nationally representative surveys exists for high-income countries, but is so far lacking for many developing countries. More work is needed to better understand cultural and socioeconomic drivers of social welfare perceptions. Comprehensive, detailed, and context-tailored studies concerning the emerging issue of animal welfare in developing countries are therefore very timely and may help facilitate the improvement of animal welfare across the developing world.

## About the Authors

**Martin Christoph Parlasca** is an agricultural economist, currently working as a senior researcher at the Center for Development Research (ZEF), Bonn University (Germany). In his research, Martin analyzes issues related to nutrition and food security in developing countries, as well as the role of livestock within sustainable food systems around the world. He holds a MSc degree in agricultural and food economics from Bonn University and a PhD in economics from Göttingen University (Germany).

**Isabel Knößlsdorfer** worked as a junior researcher at the department for International food economics and rural development at the Göttingen University (Germany), where she also completed her PhD in agricultural economics. Before that she majored in development studies and anthropology. Her research interests revolve around food security, sustainable diets and other sustainability issues, international relations, and household welfare.

**Gezahegn Alemayehu** is a research officer at International Research Institute (ILRI). Previously, he served Samara University as Associate professor. He has a PhD and MSc in veterinary public health from Addis Ababa University and a DVM degree from Jimma University. His research interest mainly focused on animal behavior and welfare, infectious disease epidemiology, and livestock reproductive technologies.

**Rebecca Doyle** is a senior lecturer of animal welfare and the Deputy Director of the Jeanne Marchig International Centre for Animal Welfare Education (JMICAWE), the University of Edinburgh, and a contributing scientist in animal welfare at International Livestock Research Institute (ILRI). Rebecca has a PhD in animal welfare from the University of New England and a Bachelor’s degree in Animal Science from the University of Sydney, and has researched animal welfare for over 14 years. Her research interests focus on understanding and improving animal welfare in diverse contexts, and the role of animal welfare in the context of sustainable livestock systems.
